# Supplementary material for: CREB Is Indispensable to KIT Function in Human Skin Mast Cells—A Positive Feedback Loop between CREB and KIT Orchestrates Skin Mast Cell Fate
Source: Cells. 2023 Dec 24;13(1):42. doi: 10.3390/cells13010042 (PMC10778115; doi:10.3390/cells13010042)
Supplement: Supplementary file 1 [file cells-13-00042-s001.zip › cells-2772761-supplementary.pdf]

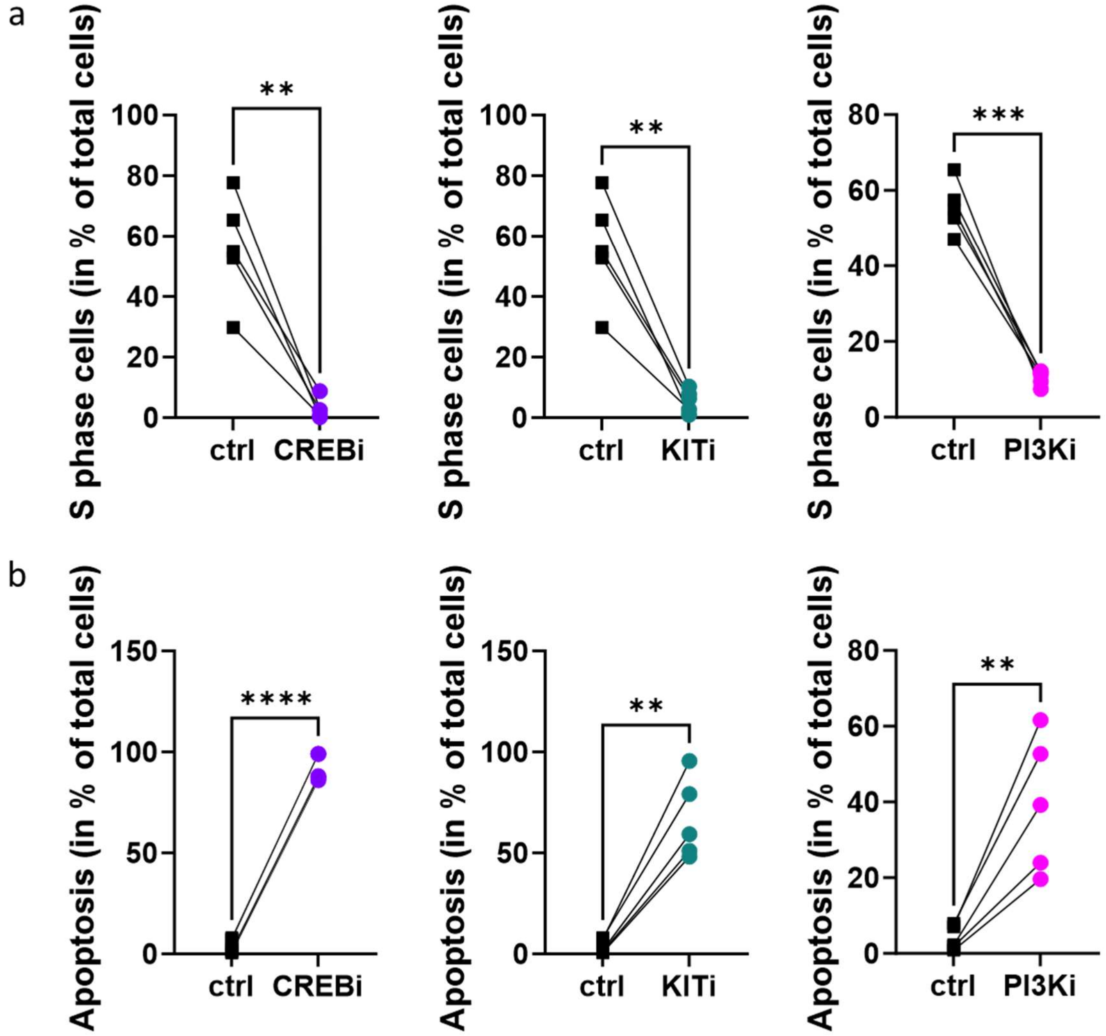

Supplementary figure S1.

Inhibition of CREB, KIT or PI3K interrupts cell cycle progression and induces apoptosis in skin MCs. MC cells were treated with either CREBi, KITi, PI3Ki or DMSO (ctrl), as detailed in Methods, and incubated in the presence of 100 ng/ml SCF and BrdU for a total of 5 days. Cells were harvested, and after application of the anti-BrdU antibody, total DNA was labelled with the 7-AAD dye. The distinct cell cycle phases were gated as given in main Figure 6. Effect of the indicated inhibitors on the proportions of S phase cells (a) and apoptotic cells (b), respectively. Note that the proportion of identifiable cells, which served as basis for the calculations, was different between treatments since barely any identifiable cells were left in the setting with CREBi (main Figure 6). n=5; **, $p < 0.01$; ***, $p < 0.001$; ****, $p < 0.0001$ with Student's t-test. This figure corresponds with main Figure 6.
